# Exploring Health‐Related Quality of Life in Children With Pulmonary Hypertension

**DOI:** 10.1002/pul2.70161

**Published:** 2025-09-28

**Authors:** Jo Wray, Sadia Quyam, Holly Clisby, Vicky Kelly, Shahin Moledina

**Affiliations:** ^1^ Heart and Lung Directorate Great Ormond Street Hospital for Children NHS Foundation Trust London UK; ^2^ Institute of Cardiovascular Science, UCL London UK; ^3^ Barts Heart and Thorax Centre Psychological Services London UK

**Keywords:** disease severity, learning disability, routine evaluation, survival

## Abstract

Pulmonary hypertension (PH) in children requires complex medical management. Health‐related quality of life (HRQoL) remains understudied in this population. During an 8‐month period children and parents attending PH outpatient appointments completed the generic PedsQL (measuring physical, emotional, social, and school functioning). Parents completed the Hospital Anxiety and Depression scale, a validated measure of anxiety and depression, about their own mental health. Clinical data were extracted from the medical notes. Analyses explored relationships between clinical factors, parental mental health and HRQoL and compared scores with published norms. Parents of 94 of 98 (96%) eligible children with PH and 48 of 54 (89%) eligible children aged ≥ 5 years completed the PedsQL. All HRQoL scores were significantly below healthy norms, with 49% scoring > 2 S.D. below normative means. Physical HRQoL was associated with disease severity and survival outcomes. Multiple regression analyses showed age, learning disability, functional class, and parental depression explained 38% of parent‐reported HRQoL variance (F(6, 86) = 7.67; *p* < 0.001) while learning disability explained 33% of child‐reported variance (F(3, 45) = 6.78; *p* < 0.001). These findings support routine HRQoL evaluation and development of disease‐specific measures for paediatric PH.

Pulmonary hypertension (PH) is a life‐limiting condition characterised by elevated pulmonary artery pressure leading to right‐sided heart failure. Recent advances in medical management and novel drug therapies have significantly improved survival rates, expanding clinical focus to include understanding disease impact on daily life, particularly health‐related quality of life (HRQoL).

Unlike the well‐documented HRQoL in adult PH populations [1, 2, 3, 4], evidence in paediatric PH remains limited. Four systematic evaluations have demonstrated consistently poorer HRQoL in children with PH compared to healthy peers [5, 6, 7, 8], though these studies were constrained by small sample sizes (25–47 patients). The relationship between disease severity and HRQoL remains unclear, with some studies showing correlation with WHO functional class [5, 7, 8] and others showing no association with parameters of disease severity or treatment [6].

Emerging evidence suggests parental well‐being may significantly influence HRQoL in this population [6] though this relationship requires further investigation. Additionally, while there is evidence of stronger agreement between child and parent‐proxy reports of HRQoL in paediatric PH compared to other chronic conditions [7], this finding stems from a single study of 18 child‐parent pairs; few studies ask children themselves, relying instead on parent‐proxy ratings of HRQoL. Notably, the impact of learning disabilities and neurodevelopmental delay, both of which are frequent co‐morbidities in paediatric PH [9], has not been systematically evaluated.

Furthermore, while HRQoL measures have demonstrated prognostic value in adult PH [4], no paediatric studies have examined the relationship between HRQoL scores and clinical outcomes. Understanding such associations could facilitate targeted interventions to improve outcomes.

To address these knowledge gaps, we analysed routinely collected child and parent‐proxy reported HRQoL data in a large cohort of children with PH. Our aims were to: 1) validate previous findings regarding HRQoL impairment in a larger cohort; 2) examine the impact of learning disability on HRQoL; 3) explore the prognostic value of HRQoL measures; and 4) investigate relationships between clinical factors, parental mental health, and HRQoL in this population.

## 1 Methods

### 1.1 Study Design

This cross‐sectional study reporting prospectively acquired HRQoL and routinely collected clinical data was conducted at Great Ormond Street Hospital, London with institutional Research and Development Office approval. Full ethical approval was not required.

### 1.2 Study Population

In the UK, specialised PH care has been centralised since 2001 to provide a consistent national approach to diagnosis and management. All children with PH are managed by a single centre that acts as the hub of the specialist paediatric PH network. Inclusion criteria for this study comprised patients established within the PH service, attending their outpatient clinic appointments between 07/04/2011 and 19/04/2012 with a confirmed PH diagnosis. Diagnoses were established through cardiac catheterisation based on established haemodynamic criteria (mean pulmonary artery pressure ≥ 20 mmHg at rest) or through comprehensive noninvasive clinical assessment including echocardiographic signs of elevated pulmonary pressure, clinical signs consistent with PH, and multidisciplinary team consensus, reflecting contemporary paediatric practice [9].

Upon clinic arrival, families received written information explaining the rationale for collecting HRQoL and parental mental health data and provided verbal consent. A psychology assistant was available to help children and/or parents complete the measures when needed. For participants with multiple clinic visits, data from the initial assessment were analysed.

### 1.3 Assessment Measures

#### 1.3.1 Health‐Related Quality of Life

The PedsQL4.0 [10, 11] was administered to parents of children aged 0–18 years and to children aged 5–18 years. This validated 23‐item generic instrument assesses four domains: physical, emotional, social, and school functioning. In addition to individual subscale scores, a psychosocial summary score and total score can be calculated. Scores range from 0 to 100, with higher scores indicating better perceived HRQoL. For infants, age specific versions were used [12]: 0–12 months (36 items) and 13–23 months (45 items), evaluating physical functioning/symptoms and emotional, social, and cognitive domains. The PedsQL4.0 has been widely used with healthy, acutely and chronically ill populations, including cardiac and respiratory patients, and there are extensive data demonstrating the internal reliability and validity of the measure.

### 1.4 Parental Mental Health

Parents completed the Hospital Anxiety and Depression Scale (HADS) [13], comprising separate 7‐item anxiety and depression subscales (scoring range: 0–21). Scores are categorised as normal (0–7), mild (8–10) moderate (11–14), and severe (15–21). Parents scoring in moderate/severe ranges were offered psychology referral.

### 1.5 Clinical Parameters

Demographic and clinical data were extracted from the medical notes, including diagnosis, WHO functional class, medications, co‐morbidities, and presence of learning disability. Learning disability was defined by syndrome diagnosis, formal developmental/cognitive testing, and/or parental report of school performance.

### 1.6 Statistical Analysis

Descriptive statistics were used to explore the study sample. One sample *t*‐tests were used to compare HRQoL scores with published healthy norms [10, 12, 14]. Chi‐squared, Mann‐Whitney, and Kruskal‐Wallis tests were used to compare PedsQL and HADs scores between diagnostic and functional status groups and to compare outcomes for those with and without a learning disability. Given the exploratory nature of the analysis, no corrections for multiple comparisons were applied.

Spearman rho bivariate correlations examined: (1) correlations between child and parent scores; (2) associations between clinical, demographic, and parental mental health variables with HRQoL scores. Differences between child and parent ratings were analysed using the Bland‐Altman Limits of Agreement analysis, supplemented by exact 95% confidence intervals. Multiple regression analyses were conducted using variables showing significant correlations (*p* < 0.05) with either child and/or parent total HRQoL to identify key predictors. Due to limited participants in individual age categories, all age groups were analysed together except when comparing to healthy norms. A Cox proportional survival model assessed association between HRQoL measures and death/transplantation outcomes.

## 2 Results

### 2.1 Study Population and Demographics

From 98 eligible children attending during the study period, 94 (96%) were enrolled, with the remaining four families declining to complete questionnaires. Parents of all 94 children (92 mothers, 2 fathers) completed questionnaires. Among 54 children eligible for self‐reporting (aged ≥ 5 years), 48 (89%) completed questionnaires, with non‐completion due to learning disability (*n* = 5) or refusal (*n* = 1) (Table 1). One parent did not complete the HADS due to time constraints.

**Table 1:** Demographic and medical data for the 94 patients.

Variable	Number
Gender: Number of males	50 (53%)
Age: mean (SD) (years)	6.95 (4.85)
Age range: (years)	0.3–16
Ethnicity: White	54 (57%)
Asian	7 (8%)
Black	7 (8%)
Mixed	3 (3%)
Not specified	23 (24%)
Distance from treating hospital: median (range) (miles)	40 (2–503)
Diagnosis of definite or probably learning disability: Idiopathic^a^	3 (19%)
CHD	10 (29%)
Left heart disease	1 (25%)
Respiratory disease	10 (37%)
Group 5	0 (0%)
WSPH diagnosis:	
Group 1	53 (56%)
IPAH	16 (17%)
PAH A‐CHD	37 (39%)
Group 2 (Left heart disease)	4 (4%)
Group 3 (respiratory disease)	27 (29%)
Group 5	10 (11%)
Treatment type: None	14 (15%)
Oral medications only	70 (75%)
Intravenous medication	10 (11%)
Number of medications: median (range)	3 (0–10)
Functional class: I	33 (35%)
II	30 (32%)
III	26 (28%)
IV	2 (2%)
Not recorded	3 (3%)

a %s refer to the proportion of each diagnostic group with definite or probably learning disability.

### 2.2 Diagnostic Groups

The cohort comprised patients across WSPH diagnostic groups. Fifty‐three patients (56%) were classified as Group 1 PH, of which 16 (17%) had idiopathic PAH and 37 (39%) had PAH associated with CHD. Four patients (4%) had Group 2 PH, while 27 patients (29%) had Group 3 PH associated with respiratory disease. The remaining 10 patients (11%) had Group 5 PH, which included underlying causes such as scimitar syndrome, segmental PH, and metabolic causes.

### 2.3 Haemodynamic Characteristics

Invasive haemodynamic confirmation of PH was available in 64/94 patients (68%), with the remainder diagnosed through comprehensive clinical assessment by the multidisciplinary team. Among catheterised patients, median mean pulmonary artery pressure (mPAP) was 32 mmHg (range 20–94 mmHg) and median pulmonary vascular resistance index was 17WU.m2 range (3–49 WU.m2).

### 2.4 Co‐Morbidities

Learning disability was present in 24 children (26%), with highest prevalence in the lung disease group (*n* = 10; 37%).

### 2.5 Pulmonary Hypertension Therapies

Sildenafil was the most commonly prescribed medication (*n* = 73, 78%) followed by bosentan (*n* = 44, 47%); 21 patients were receiving dual oral therapy. Fourteen patients (15%) required prostacylin analogue therapy: 10 patients (11%) received intravenous epoprostenol and 4 patients (4%) received inhaled illoprost.

### 2.6 Quality of Life Scores

**Figure 1:**
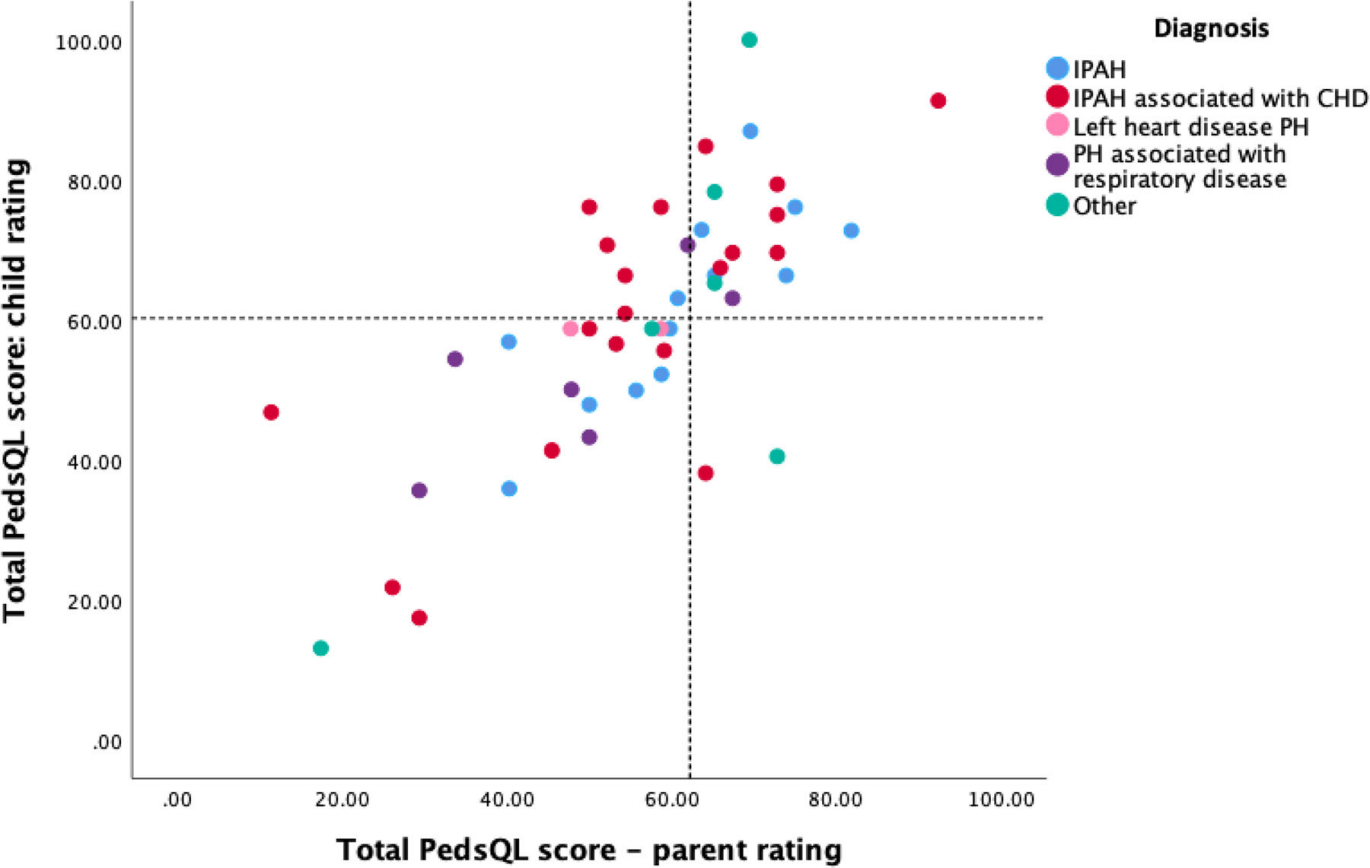
Scatterplot of Parent and Child PedsQL Total Scores by PH diagnostic group. Dotted lines represent 2 standard deviations below the mean for healthy norms. PH – pulmonary hypertension. IPAH – idiopathic pulmonary arterial hypertension. CHD – congenital heart disease.

HRQoL scores were significantly impaired across all domains compared to healthy population norms, with the exception of infants (Table 2). Effect sizes for the comparisons between the PH and healthy groups were large (> 0.8) or medium (0.5) for all domains (Table 2). Notably, 49% of children demonstrated total scores more than two standard deviations below healthy population means (Figure 1). Physical functioning scores showed the greatest impairment, especially in older age groups (Table 2). Age was significantly correlated with parent‐reported total score (*r* = −0.280, *p* = 0.007), physical (*r* = −0.333; *p* = 0.001) and psychosocial (*r* = −0.250; *p* = 0.016) summary scores but not with any child‐reported scores.

**Table 2:** Child/young person and parent PedsQL subscales and total scores (SD) for the total group, diagnostic subgroups and healthy norms.

	Idiopathic (*n* = 16)	IPAH associated with CHD (*n* = 37)	Left heart disease PH (*n* = 4)	PH associated with respiratory disease (*n* = 27)	Other (Group 5) (*n* = 10)	Total group (*n* = 94)	Healthy norms
**Infant (0–12 months)**	(*n* = 0)	(*n* = 1)	(*n* = 1)	(*n* = 0)	(*n* = 1)	(*n* = 3)	(*n* = 246)
Physical functioning	—	83.33 (−)	66.67 (−)	—	95.83 (−)	81.95 (14.63)	87.54 (11.16)
Physical symptoms	—	85.00 (−)	52.50 9−)	—	85.00 (−)	74.17 (18.76)	83.45 (10.39)
Emotional	—	81.25 (−)	75.00 (−)	—	77.08 (−)	77.78 (3.18)	76.59 (13.71)
Social	—	93.75 (−)	100.00 (−)	—	100.00 (−)	97.92 (3.61)	89.62 (14.87)
Cognitive	—	87.50 (−)	75.00 (−)	—	100.00 (−)	87.50 (12.50)	83.11 (20.65)
Physical summary	—	84.38 (−)	57.81 (−)	—	89.06 (−)	77.08 (16.85)	84.98 (9.45)
Psychosocial summary	—	85.00 (−)	80.88 (−)	—	86.25 (−)	84.04 (2.81)	80.47 (12.64)
Total score	—	84.72 (−)	69.70 (−)	—	87.50 (−)	80.64 (9.58)	82.47 (9.95)
**Infant (13–23 months)**	(*n* = 0)	(*n* = 3)	(*n* = 1)	(*n* = 6)	(*n* = 0)	(*n* = 10)	(*n* = 141)
Physical functioning	—	93.40 (8.86)	79.17 (−)	86.25 (9.49)		87.85 (9.33)	90.32 (8.96)
Physical symptoms	—	83.33 (8.78)	80.00 (−)	60.75 (18.38)		69.45 (18.22)	87.54 (9.29)
Emotional	—	80.56 (11.47)	91.67 (‐)	67.38 (25.42)		73.76 (21.59)	78.60 (12.80)
Social	—	98.33 (2.89)	85.00 (−)	78.13 (34.53)		84.88 (27.48)	91.14 (10.77)
Cognitive	—	85.18 (13.98)	83.33 (−)	52.82 (28.95)		65.59 (27.94)	84.65 (15.76)
Physical summary	—	87.96 (8.59)	79.69 (−)	76.16 (2.74)		80.49 (7.41)	88.84 (7.68)
Psychosocial summary	—	85.58 (9.47)	87.58 (−)	64.50 (22.52)		73.12 (20.64)	83.12 (11.02)
Total score	—	86.54 (3.60)	86.54 (−)	65.78 (20.11)		73.88 (18.37)	85.55 (8.74)
**Toddler (2–4 years)**	(*n* = 1)	(*n* = 10)	(*n* = 0)	(*n* = 13)	(*n* = 3)	(*n* = 27)	(*n* = 2900)
Physical functioning	78.57 (−)	58.08 (27.18)		55.82 (24.72)	42.71 (20.09)	56.05 (24.71)	89.82 (15.43)
Emotional	60.00 (−)	60.25 (22.24)		64.71 (23.54)	50.00 (13.23)	61.25 (21.48)	84.26 (14.24)
Social	100.00 (−)	69.13 (19.42)		60.42 (27.75)	51.67 (12.58)	64.28 (23.96)	88.54 (15.58)
Nursery	—	60.83 (20.81)		55.95 (27.52)	38.89 (9.62)	55.83 (22.64)	—
Psychosocial summary	80.00 (−)	64.23 (15.25)		61.92 (20.00)	48.08 (11.70)	61.91 (17.58)	86.56 (12.31)
Total score	79.41 (−)	61.73 (19.61)		59.42 (19.55)	46.03 (14.79)	59.53 (19.07)	87.86 (12.19)
**Child: 5–7 years**	(*n* = 3)	(*n* = 4)	(*n* = 1)	(*n* = 2)	(*n* = 1)	(*n* = 11)	(*n* = 1915)
Physical functioning	58.33 (23.66)	57.37 (14.73)	43.75 (‐)	65.63 (13.26)	56.25 (−)	57.79 (15.07)	86.23 (13.22)
Emotional	73.33 (25.17)	63.33 (11.55)	80.00 (‐)	60.00 (0.00)	50.00 (−)	66.00 (15.78)	78.54 (18.35)
Social	70.00 (30.00)	62.50 (17.08)	80.00 (−)	45.00 (7.07)	100.00 (−)	66.36 (22.48)	81.55 (17.51)
School	60.00 (17.32)	36.88 (17.00)	40.00 (−)	50.00 (14.14)	60.00 (−)	47.95 (16.76)	80.04 (16.55)
Psychosocial summary	67.78 (22.19)	53.15 (12.73)	66.67 (−)	51.67 (7.07)	70.00 (−)	59.63 (14.74)	80.08 (14.25)
Total score	64.49 (20.20)	54.93 (12.92)	58.70 (−)	56.52 (9.22)	65.22 (−)	59.11 (12.69)	82.22 (12.55)
**Parent of child: 5–7 years**	(*n* = 3)	(*n* = 6)	(*n* = 1)	(*n* = 4)	(*n* = 1)	(*n* = 15)	(*n* = 2314)
Physical functioning	45.83 (12.63)	66.52 (14.42)	31.25 (−)	51.02 (14.43)	43.75 (−)	54.38 (16.43)	80.11 (20.85)
Emotional	70.00 (26.46)	69.17 (17.72)	45.00 (−)	45.00 (12.91)	80.00 (−)	62.00 (20.25)	80.11 (16.12)
Social	85.00 (13.23)	78.75 (12.82)	87.50 (−)	48.75 (24.28)	80.00 (−)	72.67 (21.01)	80.28 (20.62)
School	46.67 (10.41)	52.50 (27.88)	45.00 (−)	40.00 (16.83)	70.00 (−)	48.67 (20.39)	77.29 (19.44)
Psychosocial summary	67.22 (9.77)	66.98 (15.21)	57.14 (−)	44.64 (17.32)	76.67 (−)	61.06 (16.71)	79.25 (15.44)
Total score	59.78 (9.78)	66.84 (14.71)	47.73 (−)	46.84 (15.36)	65.22 (−)	58.71 (14.90)	79.56 (16.02)
**Child: 8–12 years**	(*n* = 6)	(*n* = 8)	(*n* = 1)	(*n* = 4)	(*n* = 2)	(*n* = 21)	(*n* = 2499)
Physical functioning	48.51 (14.23)	61.72 (25.26)	62.50 (−)	38.09 (14.63)	65.63 (13.26)	53.85 (20.95)	87.98 (13.77)
Emotional	62.50 (14.40)	67.50 (22.99)	65.00 (−)	68.13 (12.81)	82.50 (10.61)	67.50 (17.25)	79.31 (18.13)
Social	74.17 (17.15)	73.75 (22.16)	50.00 (−)	55.00 (21.21)	55.00 (7.07)	67.38 (20.16)	86.15 (16.53)
School	65.83 (18.55)	63.13 (21.03)	55.00 (−)	51.25 (16.52)	72.50 (24.75)	62.14 (18.81)	81.89 (16.21)
Psychosocial summary	67.50 (14.33)	68.13 (19.75)	56.67 (−)	57.83 (16.78)	70.00 (14.14)	65.62 (16.18)	82.44 (14.11)
Total score	60.03 (14.88)	65.90 (21.20)	58.70 (−)	50.95 (15.23)	68.48 (13.83)	61.56 (17.10)	84.38 (12.85)
**Parent of child: 8–12 years**	(*n* = 7)	(*n* = 8)	(*n* = 1)	(*n* = 4)	(*n* = 2)	(*n* = 22)	(*n* = 2935)
Physical functioning	48.05 (18.05)	45.37 (25.24)	56.25 (−)	35.16 (16.41)	60.94 (6.63)	46.27 (19.90)	82.91 (20.56)
Emotional	61.90 (21.35)	43.75 (25.74)	60.00 (−)	55.00 (19.58)	67.50 (10.61)	54.47 (22.16)	79.66 (17.84)
Social	70.45 (20.52)	71.25 (24.46)	60.00 (−)	40.00 (24.49)	55.00 (7.07)	63.33 (23.65)	81.39 (20.93)
School	67.14 (17.86)	55.00 (24.49)	60.00 (−)	50.00 (7.07)	62.50 (17.68)	58.86 (17.72)	76.42 (19.41)
Psychosocial summary	66.55 (16.59)	56.61 (21.52)	60.00 (−)	48.33 (15.81)	61.67 (4.71)	58.88 (17.70)	79.16 (16.21)
Total score	60.44 (15.64)	52.73 (22.60)	58.70 (−)	43.75 (15.04)	61.41 (5.38)	54.61 (17.73)	80.48 (16.28)
**Adolescent: 13–18 years**	(*n* = 4)	(*n* = 9)	(*n* = 0)	(*n* = 0)	(*n* = 3)	(*n* = 16)	(*n* = 1066)
Physical functioning	53.01 (17.55)	52.78 (21.56)		—	52.98 (44.12)	52.87 (23.86)	88.79 (13.16)
Emotional	61.25 (11.81)	71.11 (17.99)		—	62.92 (40.32)	67.11 (20.96)	80.82 (17.75)
Social	68.60 (11.16)	53.89 (33.71)		—	46.67 (48.56)	56.21 (31.74)	89.27 (14.50)
School	67.50 (10.41)	59.44 (22.97)		—	43.33 (49.07)	58.44 (26.31)	81.20 (16.75)
Psychosocial summary	65.78 (6.79)	61.48 (21.54)		—	50.28 (44.72)	60.46 (23.50)	83.74 (13.38)
Total score	61.45 (10.14)	58.45 (20.83)		—	57.84 (22.98)	58.91 (22.69)	85.49 (12.04)
**Parent of adolescent: 13–18 years**	(*n* = 5)	(*n* = 9)	(*n* = 0)	(*n* = 0)	(*n* = 3)	(*n* = 17)	(*n* = 1281)
Physical functioning	52.41 (23.54)	47.66 (22.64)		—	53.27 (40.76)	50.19 (24.81)	83.87 (20.13)
Emotional	50.00 (12.25)	65.56 (20.22)		—	65.00 (18.03)	60.88 (18.31)	80.56 (17.81)
Social	59.00 (19.49)	56.25 (32.49)		—	56.67 (40.73)	57.19 (28.58)	84.23 (19.30)
School	78.75 (13.15)	62.50 (14.39)		—	38.33 (20.21)	62.00 (19.98)	76.96 (20.02)
Psychosocial summary	61.33 (10.50)	60.97 (19.56)		—	52.88 (25.90)	59.57 (17.56)	80.55 (15.82)
Total score	59.19 (12.45)	56.36 (18.84)		—	53.22 (31.08)	56.66 (18.45)	81.75 (15.72)

### 2.7 Parent‐Child Agreement

**Figure 2:**
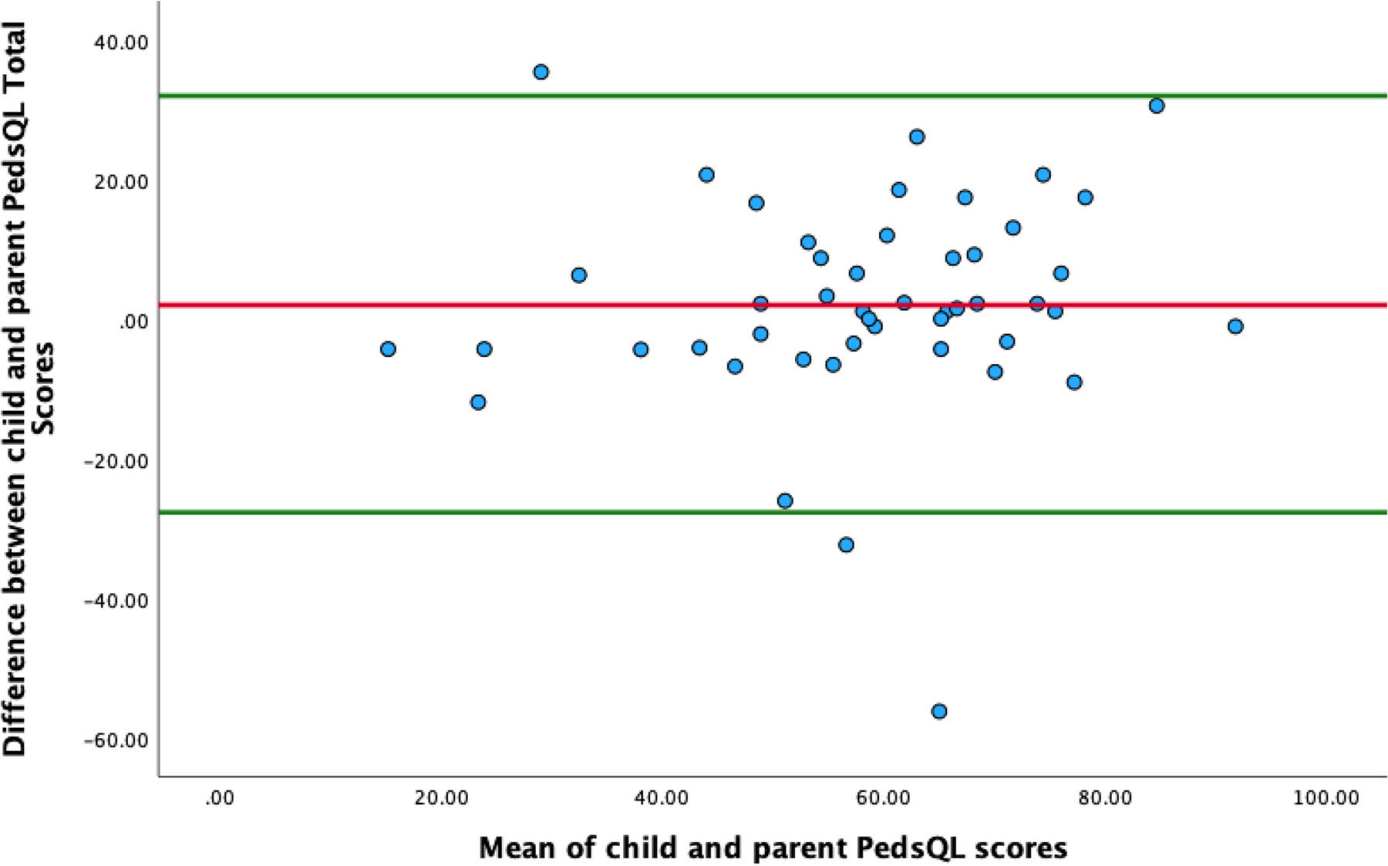
Bland‐Altman plot for child and parent reported PedsQL total score. Limits of agreement are shown as solid green lines, the red line denotes the mean difference.

Child and parent reports demonstrated strong correlation across all domains (Table 3) (physical: *r* = 0.767, emotional: *r* = 0.534, social: *r* = 0.724, and school: *r* = 0.676, all *p* < 0.01) with strongest concordance observed in the lung disease group for the physical (*r* = 0.964) and school domains (*r* = 0.895) but moderate correlations for the emotional (*r* = 0.360) and social (*r* = 0.370) domains. However, children rated their physical and emotional HRQoL as being significantly better than their parents did (*p* = 0.006 and *p* = 0.005 respectively). A Bland Altman plot of child and parent‐reported total PedsQL scores indicated no proportional bias for the overall group (B = 0.097; *p* = 0.491) (Figure 2).

**Table 3:** Parent‐child/young person correlations on PedsQL subscales, psychosocial summary scale, and total score.

	Total group (*n* = 48)	Idiopathic (*n* = 13)	IPAH associated with CHD (*n* = 21)	Left heart disease PH (*n* = 2)	PH associated with respiratory disease (*n* = 6)	Other (*n* = 6)
Physical	0.767**	0.732*	0.715**	—	0.964**	0.814*
Emotional	0.534**	0.768**	0.598**	—	0.360	0.423
Social	0.724**	0.615*	0.789**	—	0.370	0.702
School	0.676**	0.476	0.753**	—	0.895*	0.623
Psychosocial summary	0.706**	0.799**	0.753**	—	0.457	0.634
Total score	0.735**	0.786**	0.737**	—	0.756	0.726

Abbreviations: CHD, congenital heart disease; IPAH, idiopathic pulmonary arterial hypertension; PH, pulmonary hypertension.

** correlation significant at the *p* < 0.01 level.

* correlation significant at the *p* < 0.05 level.

### 2.8 Clinical Associations

WHO functional class (I: 35%, II: 32%, III: 28%, IV: 2%, not recorded 3%) demonstrated significant associations with HRQoL scores. Poorer functional class correlated with poor parent‐reported HRQoL on the physical (*r* = −0.327; *p* = 0.017) and social (*r* = −3.15; *p* = 0.022) domains and with child‐reported physical HRQoL (*r* = −0.347, *p* = 0.014). Analysis by functional class revealed significant differences in parent‐reported total HRQoL scores (F(3, 87) = 6.972; *p* < 0.001). Children in functional class I scored significantly higher than those in class III on total HRQoL (69.19 ± 18.13 vs 48.88 ± 18.45; mean difference: 20.31, 95% CI: 10.61–30.01; *p* < 0.001) physical (68.95 ± 20.21 vs 42.88 ± 21.84; mean difference: 26.07, 95% CI: 14.87–37.23; *p* < 0.001), and psychosocial score (69.53 ± 18.57 vs 52.59 ± 18.70; mean difference: 16.93, 95% CI: 7.04–26.83; *p* = 0.001).

Learning disability had a significant negative impact on HRQoL. Children with learning disability reported significantly lower scores on all self‐reported domains (*p* < 0.05). Parent ratings for these children showed significantly lower scores on all domains other than physical QoL (*p* < 0.05).

Number of medications showed significant associations with HRQoL. In univariate analysis, an increased number of medications correlated with lower child‐reported physical (*r *= −0.321; *p* = 0.028) and emotional scores (*r *= −0.347; *p* = 0.018) and parent‐reported physical HRQoL (*r *= −0.328; *p* = 0.02). However, in multiple regression analysis, number of medications was not a significant predictor in either parent or child models (Table 4).

**Table 4:** Predictors of parent‐reported and child/young person‐reported total quality of life scores.

Parent model (*n* = 93)	*B*	SE	*p*
Age	−1.12	0.38	0.004
Number of medications	−1.99	0.86	0.169
Learning disability	−10.87	3.72	0.005
Functional class	−4.29	2.11	0.047
Depression score	−1.13	0.50	0.025
Anxiety score	−0.47	0.54	0.389
	R^2^ = 0.38; F (6, 86) = 7.67; *p* < 0.001		
**Child model** (*n* = 48)			
Number of medications	−2.28	1.34	0.097
Learning disability	−20.95	5.88	0.001
Parental anxiety score	−0.78	0.52	0.097
	R^2^ = 0.33; F (3, 44) = 6.78; *p* < 0.001		

### 2.9 Parental Mental Health

While most parents reported normal (anxiety: *n* = 42, 45%; depression: *n* = 63, 68%) or mild (anxiety: *n* = 32, 34%; depression: *n* = 16, 17%) levels of psychological distress, 21% reported moderate/severe anxiety and 15% moderate/severe depression, with 9% scoring high on both scales. Parent‐reported HRQoL demonstrated significant correlations with both parental anxiety (physical: *r* = −0.357; emotional: *r* = −0.457, *p* < 0.05) and depression (emotional: *r* = −0.320, social: *r* = −0.289, *p* < 0.05)), while child‐reported scores showed no such associations for depression but there were significant correlations with parental anxiety (physical: *r* = −0.335; emotional: *r* = −0.351; *p* < 0.05). Notably, neither parental anxiety nor depression correlated with functional class, suggesting these associations were independent of disease severity.

### 2.10 Predictors of HRQoL

Multiple regression analysis identified distinct predictive models for parent and child‐reported HRQoL. For parent‐reported total HRQoL (F(6, 86) = 7.67, *p* < 0.001), a combination of age, learning disability, functional class and parental depression explained 38% of the score variance. In contrast, for child‐reported HRQoL, learning disability alone explained 33% of the score variance (F(3, 44) = 6.78, *p* < 0.001) (Table 4).

### 2.11 Survival Analysis

During a median follow‐up of 7.7 years, 24 children died and three underwent bilateral lung transplantation. Lower parent‐reported HRQoL scores were significantly associated with poorer transplant free survival (HR 0.98, CI 0.954–0.998, *p* = 0.003). This association was primarily driven by the physical domain (HR 0.971, 95% CI: 0.952–0.0990, *p* = 0.003), while the psychosocial domain showed no significant association with outcomes (HR 0.986, CI: 0.0964–1.008, *p* = 0.199). In multivariate analysis adjusting for WHO functional class, the association between physical HRQoL and outcomes was no longer significant (HR = 0.983, CI: 0.960–1.006 *p* = 0.140).

## 3 Discussion

This study represents the largest and longest evaluation of HRQoL in paediatric PH to date, with a median follow‐up of 7.74 years. Our findings definitively demonstrate that HRQoL is significantly impaired in children with PH when compared with healthy peers, with almost half of our cohort scoring more than two standard deviations below healthy population means. This magnitude of impairment exceeds previous reports from smaller studies and highlights the pervasive impact of PH on children's daily lives [5, 6, 7, 8]. While diagnostic subgroups showed comparable HRQoL scores, disease severity significantly impacted HRQoL. The substantial difference in HRQoL scores between children in WHO functional class I and III (mean difference exceeding 20 points) represents a clinically meaningful disparity that exceeds established clinically important differences for PedsQL [15]. This robust association between functional class and HRQoL resolves previous inconsistencies in the literature and establishes functional status as a key determinant of quality of life in paediatric PH [5, 6, 7].

A novel finding from our study is the marked influence of learning disability on HRQoL, accounting for 33% of variance in child‐reported scores. The highest prevalence of learning disability occurred in children with underlying lung disease (37%) and congenital heart disease (29%). These children reported significantly lower scores across multiple domains, particularly in school and social functioning. This pattern likely reflects the compound effects of cognitive challenges and physical limitations: learning disability affects academic achievement and social communication [16], while PH restricts participation in school activities. The combination may exceed standard educational support resources, creating a cycle of disadvantage as health‐related absences compound existing learning difficulties.

The relationship between parent‐reported HRQoL and clinical outcomes warrants careful interpretation. Our longitudinal analysis demonstrates that lower parent‐reported HRQoL scores, particularly in the physical domain, predict poorer freedom from death or transplantation. While both PedsQL physical domain and WHO functional class assess physical limitations, they capture distinct aspects of disease impact. Functional class represents a clinician's categorical assessment, while the PedsQL physical domain reflects detailed parental observations across multiple activities, including nuanced elements such as pain, fatigue, and frequency of limitations. Rather than suggesting a novel prognostic marker, parent‐reported physical HRQoL may complement established clinical measures in assessing disease burden.

The strong concordance between parent and child reports adds weight to previous limited evidence in paediatric PH [7]. However, children consistently rated their physical and emotional HRQoL as significantly better than their parents did, emphasising the value of obtaining both perspectives. Parents typically have greater awareness of observable physical limitations, the discordance in emotional domain scores reflects the established pattern in chronic illness where perceptions of subjective experiences differ between parent and child [17, 18]. These findings inform the ongoing discourse regarding optimal respondent selection in pediatric HRQoL assessment [19], suggesting that parent‐child discordance provides valuable insights for clinical intervention [20].

The independent association between parental mental health and child HRQoL extends recent findings [6] by demonstrating that parental depression remains a significant predictor even after controlling for disease severity. The prevalence of moderate/severe anxiety (21%) and depression (15%) in our parent cohort, the latter of which was twice as high as that seen in a UK normative sample [21], highlights the need for family‐centered care. The absence of correlation between parental mental health and disease severity suggests these psychological impacts stem from broader caregiving challenges rather than direct response to illness severity. The significant influence of parental depression on proxy‐reported HRQoL, absent in child self‐reports, reinforces the value of collecting both perspectives when evaluating quality of life in this population [22].

The association between older age in children and poorer parent‐reported HRQoL scores in physical, psychosocial, and total domains suggests increasing disease impact as children mature. While the relationship between age and HRQoL varies across chronic conditions, our findings indicate that social participation and physical limitations become more pronounced in older children with PH, potentially reflecting growing disparities with healthy peers.

Initial analyses revealed associations between medication burden and lower HRQoL scores across multiple domains. However, these associations disappeared in multiple regression models controlling for disease severity, indicating that underlying disease severity, rather than treatment burden, drives HRQoL impairment. This observation has relevance for current treatment paradigms advocating early combination therapy, though prospective studies examining the impact of treatment escalation on HRQoL, while controlling for disease severity, are needed.

This study has several limitations. Despite representing the largest cohort to date, diagnostic subgroup sizes remained relatively small, particularly regarding treatment modalities. By being inclusive in terms of patient recruitment it is important to acknowledge the heterogeneity of PH in CYP and the likelihood of other co‐morbidities contributing to reported HRQoL. The use of a generic HRQoL measure, while enabling comparison with healthy norms, may not capture all PH‐specific impacts. Additionally, while our extended follow‐up period strengthens survival analyses, the single‐center design may limit generalizability.

Future research should prioritise four key areas: (1) development of PH‐specific paediatric HRQoL measures that capture disease‐specific impacts not assessed by generic instruments; (2) longitudinal evaluation of HRQoL changes following treatment modification, including comparative analysis of how different therapeutic strategies influence HRQoL; (3) intervention studies targeting modifiable factors identified here, particularly educational support for children with learning disabilities and mental health support for parents, to determine whether addressing these factors improves HRQoL; and (4) the multi‐centre validation of the prognostic value of parent‐reported HRQoL to establish its potential role in routine clinical assessment [23].

## Author Contribution

Jo Wray designed the study, undertook the analysis, wrote the first draft of the manuscript, and approved the final manuscript. Sadia Quyam undertook part of the analysis and revised and approved the final manuscript. Holly Clisby and Victoria Kelly designed the study, collected data, and revised and approved the final manuscript. Shahin Moledina designed the study, supervised clinical data extraction, undertook the analysis, and revised and approved the final manuscript.

## Ethics Statement

This manuscript reports findings from routinely collected data so full ethical approval was not required. Ethical principles underpinned all aspects of the work. Great Ormond Street Hospital Institutional Research and Development Office approval was received for the study.

## Conflicts of Interest

The authors declare no conflicts of interest.
